# Improved immunologic response to COVID‐19 vaccine with prolonged dosing interval in haemodialysis patients

**DOI:** 10.1111/sji.13152

**Published:** 2022-03-07

**Authors:** Mathias Haarhaus, Monica Duhanes, Nataša Leševic, Bogdan Matei, Bernd Ramsauer, Rui Da Silva Rodrigues, Jun Su, Michael Haase, Carla Santos‐ Araújo, Fernando Macario

**Affiliations:** ^1^ 26705 Diaverum AB Malmö Sweden; ^2^ Department of Clinical Sciences, Intervention and Technology Division of Renal Medicine Karolinska Institutet Karolinska University Hospital Stockholm Sweden; ^3^ Karolinska University Laboratory Karolinska University Hospital Stockholm Sweden; ^4^ Medical Faculty Otto‐von‐Guericke University Magdeburg Magdeburg Germany; ^5^ Faculty of Medicine Cardiovascular Research and Development Unit Porto Portugal

**Keywords:** anti‐SARS‐CoV‐2 spike protein IgG antibodies, Coronavirus, COVID‐19, haemodialysis, SARS‐CoV‐2, vaccination

## Abstract

Vaccination against 2019 coronavirus disease (COVID‐19) can reduce disease incidence and severity. Dialysis patients demonstrate a delayed immunologic response to vaccines. We determined factors affecting the immunologic response to COVID‐19 vaccines in haemodialysis patients. All patients within a Swedish haemodialysis network, vaccinated with two doses of COVID‐19 vaccine 2‐8 weeks before inclusion, were eligible for this cross‐sectional study. Severe adult respiratory syndrome coronavirus 2 (SARS‐CoV‐2) spike protein antibody levels were determined by EliA SARS‐CoV‐2‐Sp1 IgG test (Thermo Fisher Scientific, Phadia AB) and related to clinical and demographic parameters. Eighty‐nine patients were included. Patients were vaccinated with two doses of Comirnaty (BNT162b2, 73%) or Spikevax (mRNA‐1273, 23,6%). Three patients received combinations of different vaccines. Response rate (antibody titres >7 U/mL) was 89.9%, while 39.3% developed high antibody titres (>204 U/mL), 47 (43‐50) days after the second dose. A previous COVID‐19 infection associated with higher antibody titres (median (25th‐75th percentile) 1558.5 (814.5‐3,763.8) U/mL vs 87 (26‐268) U/mL, *P* = .002), while time between vaccine doses did not differ between groups (*P* = .7). Increasing SARS‐CoV‐2 antibody titres were independently associated with increasing time between vaccine doses (B 0.241, *P* = .02), decreasing serum calcium levels (B −0.233, *P* = .007) and previous COVID‐19 (B 1.078, *P* < .001). In conclusion, a longer interval between COVID‐19 mRNA vaccine doses, lower calcium and a previous COVID‐19 infection were independently associated with a stronger immunologic vaccination response in haemodialysis patients. While the response rate was good, only a minority developed high antibody titres, 47 (43‐50) days after the second vaccine dose.

## 1 INTRODUCTION

Patients with chronic kidney disease (CKD) are especially vulnerable to infection with 2019 coronavirus disease (COVID‐19) and develop more often serious complications than other populations.^1^ Dialysis patients have therefore been granted high priority in the roll‐out of COVID‐19 vaccination campaigns in many countries. Several studies have reported an impaired humoral immunologic response to COVID‐19 vaccines^2^, ^3^ and a prolonged time to peak levels of severe adult respiratory syndrome coronavirus 2 (SARS‐CoV‐2) spike protein IgG antibodies in dialysis patients after COVID‐19 vaccination.^2^, ^4^ Optimal timing of vaccine doses has become an important topic of discussion. An improved immunologic response with prolongation of the time interval between dose one and dose two of Vaxzevria and Comirnaty has been described in non‐CKD populations.^5^, ^6^ An effect of variable time intervals between the first and second vaccine dose on the immunologic response has not yet been described in dialysis patients. We studied the effect of a prolongation of the interval between the first and second dose of COVID‐19 vaccines on the humoral immunologic response in haemodialysis patients. Most patients in the current study received two doses of mRNA vaccine (Comirnaty, BNT162b2, Pfizer/BioNTech or Spikevax, mRNA‐1273, and Moderna), but one patient received a combination of mRNA and a vector‐based vaccine (Comirnaty and Vaxzevria, ChAdOx‐1 and AstraZeneca). Manufacturers’ recommendations for the time between doses are 3 weeks for Comirnaty and 4 weeks for Spikevax. However, Swedish health authorities recommend dose intervals of 3‐7 weeks for the Comirnaty vaccine and 4‐7 weeks for the Spikevax vaccine. Thus, the time gap between vaccine doses varied for patients treated at the participating dialysis centres in the current study.

## 2 MATERIALS AND METHODS

### 2.1 Patients

The current cross‐sectional study was performed in five haemodialysis outpatient clinics in Central Sweden. All 198 maintenance haemodialysis patients were screened for inclusion. Patients who had received vaccination with two doses of COVID‐19 vaccine and timing of the second dose 2‐8 weeks prior to study start were included in the study after written informed consent. Clinical and demographic data, including routine laboratory data within ±4 weeks from blood sampling, for anti‐SARS‐CoV‐2 spike protein antibodies were retrieved from the Renal Information Management System (iRIMS), a quality registry, collecting dialysis‐related data prospectively. The study was approved by the Swedish Ethical Review Authority (2021‐01442).

### 2.2 Determination of anti‐SARS‐CoV‐2 spike protein antibodies

Blood samples for anti‐SARS‐CoV‐2 spike protein IgG antibodies were collected before a haemodialysis session. Titres of anti‐SARS‐CoV‐2 IgG antibodies in serum were determined using the EliA SARS‐CoV‐2‐Sp1 IgG test (Thermo Fisher Scientific, Phadia AB). The assay was performed according to the manufacturer's instructions. Results <7 U/mL were considered negative and antibody titres >204 U/mL were considered strongly positive according to manufacturer's definition.

### 2.3 Statistics

Data are presented as median (25th‐75th percentiles), frequencies or percentages, if not stated otherwise. A P‐value below 0.05 was considered significant. Patients with a previous COVID‐19 diagnosis were compared with COVID‐19 naïve patients. Furthermore, we compared patients according to the type of vaccine they had received. We applied the independent‐samples median test for paired comparisons and for multiple comparisons with the Bonferroni correction for post hoc analyses. For correlation and regression analyses, continuous variables were standardized with outliers replaced by three standard deviations. Univariate correlations were calculated using Spearman's Rho correlation coefficient, and multivariate analyses were performed using linear regression. Covariates for the determination of anti‐SARS‐CoV‐2 spike protein antibody levels 2‐8 weeks after the second vaccination were chosen, based on a *P* < .2 in univariate correlations and/or clinical relevance. Missing data were excluded pairwise or listwise. Sensitivity analyses were performed, including also patients with vaccination interval outside of the recommended range.

## 3 RESULTS

### 3.1 Patient characteristics

Of the 198 screened patients, 98 had received 2 vaccine doses, with the last dose 2‐8 weeks before inclusion. Two patients refused consent. Of 96 patients with two doses of COVID‐19 vaccine, who consented to participate in the current study, 4 patients were excluded due to unreliable anti‐SARS‐CoV‐2 spike protein antibody results and 3 patients were excluded, since the interval between the first and second vaccine dose was outside of the range recommended by Swedish authorities. Demographic and clinical data of the remaining 89 patients are listed in Table 1.

**TABLE 1:** Baseline demographic and clinical parameters

Continuous parameters	Total (N)	Median (25%‐75%)
Age (years)	89	72 (63‐80)
Dialysis vintage (months)	89	45 (23‐77)
BMI (kg/m²)	83	25.8 (23.1‐31.2)
nPCR (g/kg/day)	81	0.99 (0.85‐1.16)
Dry weight (Kg)	84	75.0 (65.5‐92.9)
IDBWG (% of dry weight)	83	2.9 (2.1‐3.6)
Kt/V	81	1.8 (1.5‐2.0)
Weekly treatment time (minutes)	85	720 (720‐810)
Mean arterial pressure (mmHg)	83	92 (86‐101)
Diastolic blood pressure (mmHg)	84	71 (63‐79)
Systolic blood pressure (mmHg)	84	136 (121‐146)
Haemoglobin (g/L)	85	112 (104‐116)
Sodium (mmol/L)	85	139.0 (137.5‐141.0)
Potassium (mmol/L)	85	4.9 (4.6‐5.3)
Calcium (mmol/L)	85	2.22 (2.08‐2.29)
Phosphate (mmol/L)	84	1.5 (1.2‐1.8)
Interval second vaccination to sample (days)	89	47 (43‐50)
Vaccination interval (days)	89	24 (21‐28)
Anti‐SARS‐CoV‐2 s‐protein antibody (U/mL)	89	114.0 (27.0‐430.5)

Categorical parameters	Total (N)	N (%)
Type of COVID‐19 vaccine	89	
‐ BNT162b2		65 (73)
‐ mRNA‐1273		21 (23.6)
‐ 2 different vaccines		3 (3.4)
Sex ‐ Female	89	35 (39)
Type of access	87	
‐ Arteriovenous fistula		51 (57.3)
‐ Arteriovenous graft		19 (21.3)
‐ Central dialysis catheter		17 (19.1)
Type of haemodialysis treatment	89	
‐ Hig‐flux haemodialysis		29 (32.6)
‐ Post‐dilution haemodiafiltration		55 (61.8)
‐ Predilution haemodiafiltration		5 (5.6)

Abbreviations: BMI, body mass index; COVID‐19, 2019 coronavirus disease; IDBWG, inter‐dialytic body weight gain; Kt/v, measure of dialysis adequacy; nPCR, normalized protein catabolic rate; SARS‐CoV‐2, severe adult respiratory syndrome coronavirus 2.

### 3.2 Vaccination against COVID‐19

Patients were either vaccinated with two doses of Comirnaty (N = 65), two doses of Spikevax (N = 21) or combinations of different vaccines (N = 3). Median interval between vaccine dose 1 and dose 2 was 24 (minimum 21 – maximum 45) days, and median anti‐SARS‐CoV‐2 spike protein antibody level was 114.0 (27.0‐430.5) U/mL. While median time between the first and second doses differed between groups (Comirnaty 21 (minimum 21 – maximum 42) days, Spikevax 28 (minimum 28 – maximum 35) days, vaccine combinations 30 (minimum 22 – maximum 45) days, *P* < .001), no significant difference was found for anti‐SARS‐CoV‐2 spike protein antibody levels (Comirnaty 95 (minimum 0 – maximum 76 330) U/mL, Spikevax 185 (minimum 0 – maximum 3445) U/mL, vaccine combinations 219 (minimum 22 – maximum 1623) U/mL, *P* = .6). Two patients received combinations of Comirnaty and Spikevax (45 days, 1623 U/mL and 30 days, 22 U/mL), and one patient received Spikevax and Vaxzevria (28 days, 219 U/mL). Median time between last vaccine dose and blood sampling was 47 (43‐50) days for all patients with no significant differences between types of vaccines used (*P* = .08).

### 3.3 Determinants of anti‐SARS‐CoV‐2 spike protein antibodies

Response rate was 89.9%, while 39.3% developed a strong antibody response. A previous COVID‐19 infection (N = 10; 2 Spikevax, 8 Comirnaty) was clearly associated with higher antibody titres after vaccination (1558.5 (814.5‐3763.8) U/mL vs. 87 (26‐268) U/mL, *P* = .002), while time between the first and second vaccine doses did not differ between patients with and without a previous COVID‐19 infection (28 (21‐30) vs 23 (21‐28) days, *P* = .7). Univariate correlations of demographic and clinical parameters with circulating anti‐SARS‐CoV‐2 spike protein antibodies are listed in Table 2. Multivariate analysis of all patients revealed independent associations of an increasing time interval between the first and second vaccine doses, a history of COVID‐19, and lower serum calcium levels with a stronger immunologic vaccine response (Table 3). Sensitivity analyses, including also patients with vaccination intervals outside of the recommended range, revealed similar results (Tables S1 and Table S2).

**TABLE 2:** Correlation coefficients for univariate correlations with anti‐SARS‐CoV‐2 spike protein IgG antibodies

	N	Correlation coefficient	*P*
Blood flow (mL/min)	86	−.057	.60
Diastolic blood pressure (mmHg)	84	.115	.30
Systolic blood pressure (mmHg)	84	.118	.29
Dialysate flow (mL/min)	85	.016	.88
Haemoglobin (g/L)	85	−.129	.24
Sodium (mmol/L)	85	−.269	.01
Potassium (mmol/L)	85	.127	.25
Calcium (mmol/L)	85	−.340	.001
Phosphate (mmol/L)	84	−.026	.82
Venous standard Bicarbonate (mmol/L)	76	−.004	.98
Treatment time (minutes/week)	85	.146	.183
Mean arterial pressure (mmHg)	83	.137	.22
Pulse pressure (mmHg)	83	.051	.65
Body mass index (/kgm²)	83	.177	.11
Interdialytic body weight gain (kg)	83	.179	.105
Kt/V	81	−.188	.09
Normalized protein catabolic rate (g/kg/day)	81	−.082	.47
Vaccination interval (days)	89	.195	.07
Interval 1st vaccine dose to sample (days)	89	.142	.18
Interval 2nd vaccine dose to sample (days)	89	.057	.60
Age (years)	89	−.083	.44
Dialysis vintage (months)	89	−.157	.14

Note

SARS‐CoV‐2, severe adult respiratory syndrome coronavirus 2; IgG, immunoglobulin G; Kt/v, measure of dialysis adequacy.

**TABLE 3:** Multivariate analysis of predictors of a stronger SARS‐CoV‐2 spike protein IgG response to COVID‐19 vaccines

	Regression coefficient (95% CI)	*P*
(Constant)	0.015 (−0.507 to 0.537)	.95
Type of vaccine	−0.194 (−0.566 to 0.179)	.30
Sodium (1 SD)	−0.154 (−0.309 to 0.001)	.051
Calcium (1 SD)	−0.233 (−0.4 to −0.067)	.007
Treatment time (1 SD)	−0.05 (−0.234 to 0.133)	.586
Body mass index (1 SD)	0.104 (−0.086 to 0.294)	.279
IDBWG (1 SD)	0.118 (−0.042 to 0.278)	.145
Kt/V (1 SD)	0.009 (−0.159 to 0.177)	.915
Vaccine interval (1 SD)	0.241 (0.039 to 0.443)	.02
Interval 1st dose to sample (1 SD)	0.027 (−0.142 to 0.197)	.750
Previous COVID‐19	1.078 (0.56 to 1.596)	<.001

Abbreviations: COVID‐19, 2019 coronavirus disease; IDBWG, inter‐dialytic body weight gain; IgG, immunoglobulin G; Kt/v, measure of dialysis adequacy; SARS‐CoV‐2, severe adult respiratory syndrome coronavirus 2.

## 4 DISCUSSION

The current study demonstrates for the first time an independent association of a longer interval between two COVID‐19 vaccine doses with a stronger anti‐SARS‐CoV‐2 spike protein IgG antibody response in haemodialysis patients. Our findings are in line with previous reports of an improved immunologic response to a delay of the second vaccine dose in non‐CKD patients.^5^, ^6^ We have previously demonstrated a reduced antibody response in haemodialysis patients without prior COVID‐19 infection, compared with healthy controls or patients with a previous COVID‐19 infection, 2 weeks after a single dose of Comirnaty or Vaxzevria vaccine.^7^ However, seroconversion rates after two vaccine doses in haemodialysis patients are similar to those seen in general populations, albeit after a longer time interval.^2^, ^3^, ^4^ We hypothesize that the improved immunologic response associated with a longer interval between the first and second vaccine doses in the current study may be due to a delayed immunologic response, possibly related to differences in the immune system of haemodialysis patients, that might affect the affinity maturation process and the magnitude of the antibody response.

Prolongations of vaccination intervals up to several months have been suggested by health administrations, not only to achieve an improved immunologic response, but also to meet vaccine shortages. However, recently, such long prolongations have been questioned, due to the risk of insufficient protection after a single vaccine dose, especially against rapidly spreading new variants.^8^ Although antibodies are just one of several components implicated in protective immunity to SARS‐CoV‐2, increasing evidence shows that measured immunity correlates with clinical protection against symptomatic and asymptomatic SARS‐CoV‐2 infection.^9^ It is thus reassuring that we saw an improved humoral immunologic response in the current study in spite of a short prolongation of the interval between vaccine dose 1 and dose 2, which was at maximum 45 days.

Seroconversion rate was 89.9% with no significant difference in antibody levels between Comirnaty, Spikevax or combinations of different vaccines. This finding is in line with previous reports.^2^, ^3^ The fact, that a considerably lower proportion of patients achieved a strong antibody response in the current and in previous studies^2^, ^3^ suggests, that dialysis patients may need a third booster dose, to achieve high antibody titres. Interestingly, a recent report from a French dialysis cohort demonstrated an impressive improvement of antibody titres one month after a third vaccine dose in haemodialysis patients with a sub‐optimal immunologic response after 2 doses of the Comirnaty vaccine.^10^ We found a strong association between a previous COVID‐19 infection and a more robust immunologic response to COVID‐19 vaccines, which confirms earlier reports.^7^, ^11^ The most likely mechanism could be a rapid re‐activation of the immunological memory to SARS‐CoV‐2 by COVID‐19 vaccines.^7^, ^12^ However, the accelerated decline in serum levels of anti‐SARS‐CoV‐2 antibodies after a previous COVID‐19 infection in dialysis patients^13^ may indicate that a single vaccine dose for patients with a previous COVID‐19 infection, as suggested for general populations by Mazzoni et al,^12^ may not be sufficient in dialysis patients.

Experimental and clinical evidence for a modulation of vaccine responses by serum calcium in advanced CKD is few and divergent. A high‐calcium diet has been shown to improve the immune response to tetanus vaccine in uremic rats.^14^ On the contrary, treatment of dialysis patients with paricalcitol did not improve the response to a standard booster dose of hepatitis B vaccine, in spite of increasing serum calcium levels.^15^ Thus, our finding of an association of lower serum calcium levels with a stronger immune response to COVID‐19 vaccines is hypothesis generating, but needs to be confirmed by additional studies.

Limitations of the current study are the observational and cross‐sectional character, which does not allow any causative deductions. Further limitations are the relatively small number of participants, the lack of clinical data on SARS‐CoV‐2 infections after vaccination and the lack of baseline samples for determination of anti‐SARS‐CoV‐2 spike protein antibodies. The timing of the blood samples in the current study is somewhat later than the expected early peak of antibody levels after the second dose of COVID‐19 vaccine in general populations.^16^ However, it is in accordance with the peak AB responses in a previous kinetic study in haemodialysis patients^4^ and a systematic review of antibody studies in haemodialysis patients.^2^

In conclusion, we demonstrate an independent association of a longer time interval between the first and second dose of COVID‐19 vaccines with a better immunologic response in maintenance haemodialysis patients. Our findings may aid in future development of vaccination strategies for dialysis patients in face of variable access to COVID‐19 vaccines.

## CONFLICT OF INTEREST

MaH, MD, NL, BM, BR, MiH, CS and FM are employees of Diaverum. MaH is clinical advisory board member of Resverlogix, unrelated to the submitted work. RDR and JS have nothing to declare.

## ETHICAL APPROVAL

Written informed consent was obtained from all subjects. The study protocol complied with the Declaration of Helsinki and its amendments. The study was approved by the Swedish Ethical Review Authority (2021‐01442).

## Supporting information

Table S1‐S2

## Data Availability

The data that support the findings of this study are available from the corresponding author upon reasonable request.
