# Supplementary material for: Improved immunologic response to COVID‐19 vaccine with prolonged dosing interval in haemodialysis patients
Source: Scand J Immunol. 2022 Mar 7;95(5):e13152. doi: 10.1111/sji.13152 (PMC9115353; doi:10.1111/sji.13152)
Supplement: Supplementary file 1 — Table S1‐S2 [file SJI-95-e13152-s001.doc]

**Improved immunologic response to COVID-19 vaccine with prolonged dosing interval in hemodialysis patients**

Mathias Haarhaus^1,2^, Monica Duhanes^1^, Nataša Leševic^1^, Bogdan Matei^1^, Bernd Ramsauer^1^, Rui Da Silva Rodrigues^3^, Jun Su^3^, Michael Haase^1,4^, Carla Santos- Araújo^1,5^, Fernando Macario^1^

^1^ Diaverum AB, Hyllie Boulevard 39, 215 37 Malmö, Sweden

^2^ Department of Clinical Sciences, Intervention and Technology, Division of Renal Medicine, Karolinska Institutet, Karolinska University Hospital, Stockholm, Sweden

^3^ Karolinska University Laboratory, Karolinska University Hospital, 171 76 Stockholm, Sweden

^4^ Medical Faculty, Otto-von-Guericke University Magdeburg, Magdeburg, Germany

^5^ Faculty of Medicine, Cardiovascular Research and Development Unit, Porto, Portugal

*Supplementary Material*

Supplementary Table 1. Correlation Ccoefficients for univariate correlation with anti-SARS-CoV-2 spike protein IgG antibodies, patients with all vaccination intervals included

|  | N | Correlation Coefficient | P |
| --- | --- | --- | --- |
| Blood flow (mL/min) | 89 | -0.054 | 0.62 |
| Diastolic blood pressure (mmHg) | 87 | 0.104 | 0.34 |
| Systolic blood pressure (mmHg) | 87 | 0.077 | 0.48 |
| Dialysate flow (mL/min) | 88 | 0.02 | 0.85 |
| Hemoglobin (g/L) | 88 | -0.133 | 0.22 |
| Sodium (mmol/L) | 88 | -0.271 | 0.01 |
| Potassium (mmol/L) | 88 | 0.094 | 0.39 |
| Calcium (mmol/L) | 88 | -0.352 | 0.001 |
| Phosphate (mmol/L) | 87 | -0.032 | 0.77 |
| Venous standard Bicarbonate (mmol/L) | 78 | -0.009 | 0.94 |
| Treatment time (minutes/week) | 88 | 0.158 | 0.14 |
| Mean arterial pressure (mmHg) | 86 | 0.108 | 0.32 |
| Pulse pressure (mmHg) | 86 | 0.017 | 0.88 |
| Body mass index (/kgm²) | 85 | 0.186 | 0.09 |
| Interdialytic body weight gain (kg) | 85 | 0.182 | 0.10 |
| Kt/V | 83 | -0.187 | 0.09 |
| Normalized protein catabolic rate (g/kg/day) | 83 | -0.078 | 0.48 |
| Vaccination interval (days) | 92 | 0.181 | 0.08 |
| Interval 1^st^ vaccine dose to sample (days) | 92 | 0.147 | 0.16 |
| Interval 2^nd^ vaccine dose to sample (days) | 92 | 0.066 | 0.53 |
| Age (years) | 92 | -0.095 | 0.37 |
| Dialysis vintage (months) | 92 | -0.138 | 0.19 |

Supplementary Table 2. Linear regression analysis of predictors of anti-SARS-CoV-2 spike protein IgG antibodies, patients with all vaccination intervals included

|  | Regression coefficient (95% CI) | P |
| --- | --- | --- |
| (Constant) | -0.050 (-0.547 – 0.448) | 0.8 |
| Type of vaccine | -0.130 (-0.472 – 0.213) | 0.5 |
| Sodium (1 SD) | -0.147 (-0.3 – 0.006) | 0.06 |
| Calcium (1 SD) | -0.191 (-0.348 – -0.033) | 0.02 |
| Treatment time (1 SD) | -0.020 (-0.207 – 0.167) | 0.8 |
| Body mass index (1 SD) | 0.078 (-0.114 – 0.271) | 0.4 |
| Interdialytic body weight gain (1 SD) | 0.111 (-0.047 – 0.270) | 0.2 |
| Vaccination interval (1 SD) | 0.213 (0.023 – 0.404) | 0.03 |
| KtV_(1 SD) | 0.015 (-0.155 – 0.186) | 0.9 |
| Interval 1^st^ vaccine dose to sample (1 SD) | 0.024 (-0.147 – 0.196) | 0.8 |
| Previous COVID-19 | 1.069 (0.546 – 1.593) | <0.001 |
| Dialysis vintage (1 SD) | -0.086 (-0.267 – 0.096) | 0.4 |

SARS-Co-V-2, severe adult respiratory syndrome coronavirus 2; CI, confidence interval; SD, standard deviation
